# Seasonal activity of *Dermacentor reticulatus* ticks in the era of progressive climate change in eastern Poland

**DOI:** 10.1038/s41598-021-99929-y

**Published:** 2021-10-14

**Authors:** Zbigniew Zając, Joanna Kulisz, Aneta Woźniak, Katarzyna Bartosik, Adil Khan

**Affiliations:** 1 Department of Biology and Parasitology, Medical University of Lublin, Radziwiłłowska 11 st, 20-080 Lublin, Poland; 2 Department of Zoology, Abdul Wali Khan University, Mardan, Pakistan

**Keywords:** Ecology, Ecology

## Abstract

*Dermacentor reticulatus* ticks are one of the most important vectors and reservoirs of tick-borne pathogens in Europe. Changes in the abundance and range of this species have been observed in the last decade and these ticks are collected in areas previously considered tick-free. This may be influenced by progressive climate change. Eastern Poland is an area where the local population of *D. reticulatus* is one of the most numerous among those described so far. At the same time, the region is characterized by a significant increase in the mean air temperature in recent years (by 1.81 °C in 2020) and a decrease in the average number of days with snow cover (by 64 days in 2020) and in the number of days with frost (by 20 days in 2020) on an annual basis compared to the long-term average. The aim of our research was to investigate the rhythms of seasonal activity and the population size of *D. reticulatus* in the era of progressive climate change. To this end, questing ticks were collected in 2017–2020. Next, the weather conditions in the years of observation were analyzed and compared with multi-year data covering 30 years preceding the study. The research results show that, in eastern Poland, there is a stable population of *D. reticulatus* with the peak of activity in spring or autumn (up to a maximum of 359 individuals within 30 min of collection) depending on the year of observation. Ticks of this species may also be active in winter months. The activity of *D. reticulatus* is influenced by a saturation deficit.

## Introduction

*Dermacentor reticulatus* ticks, along with *D. marginatus* and *Ixodes ricinus*, are the most widely distributed tick species in Europe^1,2^. Due to the wide spectrum of vector-borne pathogens, including tick-borne encephalitis virus, bacteria *Anaplasma* spp., *Rickettsia* spp., spirochetes *Borrelia* spp., and piroplasmid apicomplexan parasites *Babesia canis, B. caballi,* and *Theileria equi*, ticks of this species should be regarded as ectoparasites of great epidemic importance^3–5^. In addition, despite only sporadic attacks on humans^6^, *D. reticulatus* ticks feeding on many species of vertebrate animals^4,7^ constitute an important link in the transmission and maintenance of tick-borne pathogens in the environment, thus posing a real threat to public health^3^.

In recent years, changes in the range of *D. reticulatus* have been observed both on the European continent and locally in many countries^8^, e.g. the range of this species in Germany has significantly expanded over the last 50 years^9^. The northwest range of *D. reticulatus* in Europe reaches the area of the British Isles^10^. In the north of the continent, specimens of this species were collected from migratory birds^11^ and domestic animals in Scandinavia^12^. In the south, local island populations of *D. reticulatus* were described in the Iberian^13^ and Apennine^14^ peninsulas. Especially many new sites of *D. reticulatus* have been described in Central European countries^15–19^. Against this background, the case of Poland is particularly interesting, where there is a progressive expansion of two geographically separated (to a lesser extent) populations of *D. reticulatus*, i.e. Eastern and Western European^20^. Moreover, the local populations of *D. reticulatus* occurring here, especially in the eastern part of the country, are among the most numerous that have been discovered so far^21^.

The changes in the distribution range of *D. reticulatus* observed in recent years, the number of local populations of this species, and the changes in the dynamics of their seasonal activity are most likely the result of a number of factors, mainly including the progressive warming of the climate^1,8,22^. Temperature and relative air humidity are critical factors influencing the seasonal activity and host-seeking activity of *D. reticulatus*^23,24^.

In recent years, the mean annual air temperature of land and oceans in Europe has increased by 1.16°C^25^. In the area of the dense occurrence of *D. reticulatus* on the European continent, warmer and milder winters as well as hot summers are observed. For instance, the winter season 2019/2020 was the first in the history of meteorological measurements in eastern Poland with only 2 days in the entire calendar winter period with the daily maximum temperature below 0 °C (long-term average: 42 days) and the snow cover throughout the season remained only for 6 days (long-term average: 62 days)^26^.

Climate warming contributes to an increase in the range of potential tick hosts, primarily by increasing their range towards the north^27^. It also influences the characteristics of agricultural crops, and largely contributes to extension of fallow lands, creating ecological types of habitats preferred by *D. reticulatus*^28,29^.

The aim of our research was to investigate the rhythms of seasonal activity and the population size of *D. reticulatus* in eastern Poland in the era of progressive climate change.

## Results

### Weather conditions

**Figure 1:**
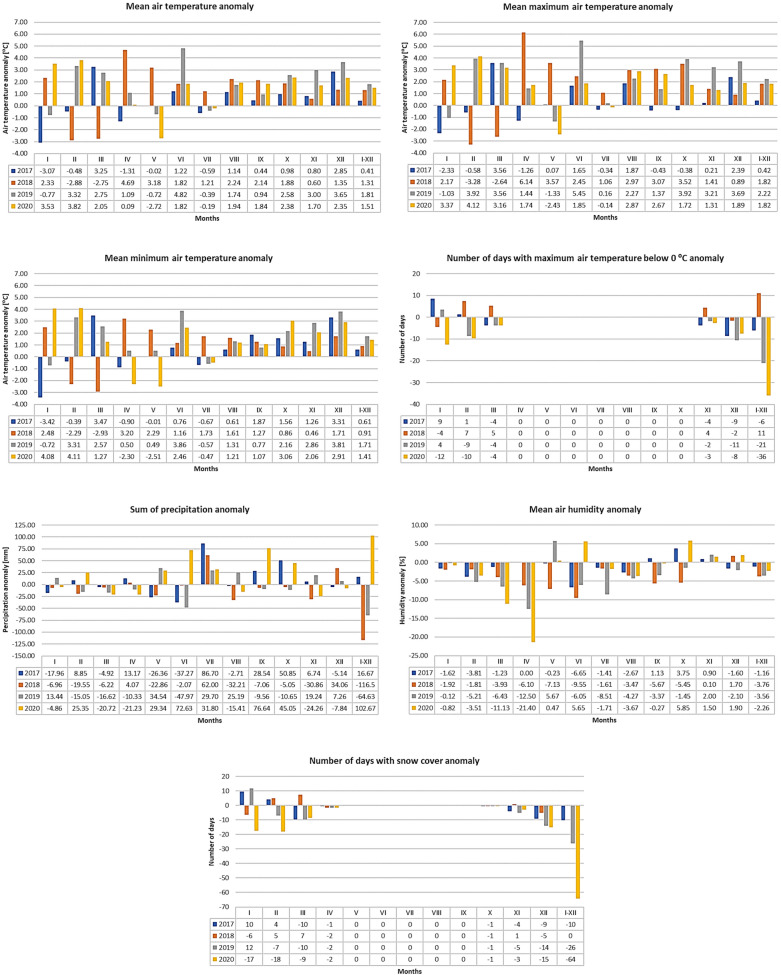
Anomalies of weather conditions in 2017–2020 compared to the multi-year reference period 1987–2016.

In the years of the study on the activity of *D. reticulatus*, the mean annual air temperature ranged from 8.4 °C to 9.8 °C (Table 1). This means a statistically significant (F = 27.7770, p = 0.0002) increase in the annual mean values relative to the reference period (1987–2016) by 0.41 °C and 1.81 °C, respectively (Fig. 1). Statistically significant positive anomalies were also observed in the mean maximum air temperature (increase by 0.42–2.22 °C) (F = 33.7516, p = 0.0001) and the mean minimum air temperature (increase by 0.61–1.71 °C) (F = 20.8825, p = 0.0008) (Fig. 1). Days with the maximum air temperature below 0 °C were noted only from November to January of each study year. In turn, in the period 1987–2016, they were usually reported from November to March (Table 1). In comparison with the long-term average value in the previous 30 years, the number of days with the maximum air temperature below 0 °C was by 11 higher in 2018, by 6 lower in 2017, by 21 lower in 2019, and by 36 lower in 2020 (Fig. 1), but these periods did not differ significantly (F = 3.4181, p = 0.0915).

**Table 1:** Comparison of the mean values of weather parameters for the reference period (1987–2016) and in the years of tick collection (2017–2020).

Years	Months												
	I	II	III	IV	V	VI	VII	VIII	IX	X	XI	XII	I–XII
**Mean air temperature [°C]**													
1987–2016	− 2.6	− 1.2	2.1	8.3	13.5	16.4	18.6	17.9	13.16	7.9	2.9	− 1.2	8.0
2017	− 5.7	− 1.7	5.4	7.0	13.5	17.7	18.1	19.1	13.2	8.9	3.7	1.6	8.4
2018	− 0.0.3	− 4.1	− 0.6	13.0	16.7	18.3	19.9	20.2	13.6	9.8	3.5	0.1	9.3
2019	− 3.4	2.1	4.9	9.4	12.8	21.3	18.3	19.7	15.3	10.5	5.9	2.4	9.8
2020	0.9	2.6	4.2	8.4	10.8	18.3	18.5	19.9	14.1	10.3	4.6	1.1	9.5
**Mean maximum air temperature [°C]**													
1987–2016	− 0.2	1.8	6.2	13.6	19.3	21.2	24.3	23.7	18.2	12.2	5.7	1.0	12.3
2017	− 2.5	1.2	9.8	12.1	19.1	23.5	24.0	25.6	17.8	11.9	5.9	3.4	12.7
2018	2.0	− 1.5	3.6	19.5	22.6	24.3	25.4	26.7	21.3	15.8	7.1	1.9	14.1
2019	− 1.2	5.7	9.8	14.8	17.7	27.3	24.5	26.0	19.6	16.2	8.9	4.7	14.5
2020	3.2	5.9	9.4	15.1	16.6	23.7	24.2	26.6	20.9	14.0	7.0	2.9	14.1
**Mean minimum air temperature [°C]**													
1987–2016	− 5.1	− 4.0	− 1.4	3.7	8.3	11.3	13.4	13.0	8.8	4.3	0.4	− 3.6	4.1
2017	− 8.5	− 4.4	2.1	2.8	8.3	12.1	12.8	13.4	10.7	5.9	1.7	− 0.3	4.7
2018	− 2.6	− 6.3	− 4.3	6.9	10.6	12.5	15.2	14.4	10.1	5.2	0.9	− 1.9	5.0
2019	− 5.8	− 0.7	1.2	4.2	8.8	15.2	12.9	14.1	9.6	6.5	3.3	0.2	5.8
2020	− 1.0	0.1	− 0.1	1.4	5.8	13.8	13.0	14.0	9.9	7.4	2.5	− 0.7	5.5
**Number of days with maximum air temperature below 0 °C (mean for 1987–2016)**													
1987–2016	13	10	4	0	0	0	0	0	0	0	4	12	42
2017	22	11	0	0	0	0	0	0	0	0	0	3	36
2018	9	17	9	0	0	0	0	0	0	0	8	10	53
2019	17	1	0	0	0	0	0	0	0	0	2	1	21
2020	1	0	0	0	0	0	0	0	0	0	1	4	6
**Total sum of precipitation [mm] (mean for 1987–2016)**													
1987–2016	32.6	30.7	38.7	42.6	67.0	67.5	83.8	57.8	59.1	41.5	39.4	34.4	595.3
2017	14.7	39.6	33.8	55.8	40.6	30.2	86.7	55.1	87.6	92.4	46.2	29.3	612.0
2018	25.7	11.2	32.5	46.7	44.1	65.4	62.0	25.6	52.0	36.5	8.6	68.5	478.8
2019	46.1	15.7	22.1	32.3	101.5	19.5	29.0	83.0	49.5	30.9	58.7	41.7	530.7
2020	27.8	56.1	18.0	21.4	96.3	140.1	31.0	42.4	135.7	86.6	15.2	26.6	698.0
**Mean relative air humidity [%]**													
1987–2016	87.8	85.7	79.3	72.0	72.8	75.5	75.0	74.4	80.9	83.8	88.8	89.4	80.5
2017	86.2	81.9	78.1	72.0	72.6	68.9	73.6	71.7	82.0	87.6	89.7	87.8	79.3
2018	85.9	83.9	75.4	65.9	65.7	66.0	73.4	70.9	75.2	78.4	88.9	91.1	76.7
2019	87.7	80.5	72.9	59.5	78.5	69.5	66.5	70.1	77.5	82.4	90.8	87.3	76.9
2020	87.0	82.2	68.2	50.6	73.3	81.2	73.3	70.7	80.6	89.7	90.3	91.3	78.2
**Number of days with snow cover (mean for 1987–2016)**													
1987–2016	18	18	11	2	0	0	0	0	0	1	5	16	70
2017	28	22	1	1	0	0	0	0	0	0	1	7	60
2018	12	23	18	0	0	0	0	0	0	0	6	11	70
2019	30	11	1	0	0	0	0	0	0	0	0	2	44
2020	1	0	2	0	0	0	0	0	0	0	2	1	6

The annual rainfall in the analyzed period ranged from 478.8 mm in 2018 (anomaly − 102.67) to 698.0 mm in 2020 (anomaly + 102.67) (Table 1, Fig. 1). However, these differences were not significant (F = 0.099, p = 0.7581). Along with the increase in the mean temperature in 2017–2020, there was a decrease in the mean relative air humidity (to  − 21.40% in April 2020) (Fig. 1).

In 2017–2020, there was a statistically significant (F = 4.9836, p = 0.0473) decrease in the number of days with snow cover, especially in November–December. In the whole 2020, only 6 days with snow cover were recorded versus the multi-year average of 70 days (Table 1, Fig. 1).

### Seasonal activity and abundance of *D. reticulatus*

Throughout the study period, a total of 5768 adult *D. reticulatus* specimens, including 3072 females and 2718 males, were collected (Supplementary Table S1), but no significant difference between the sexes was observed (Z = 0.53392, p = 0.59612).

**Figure 2:**
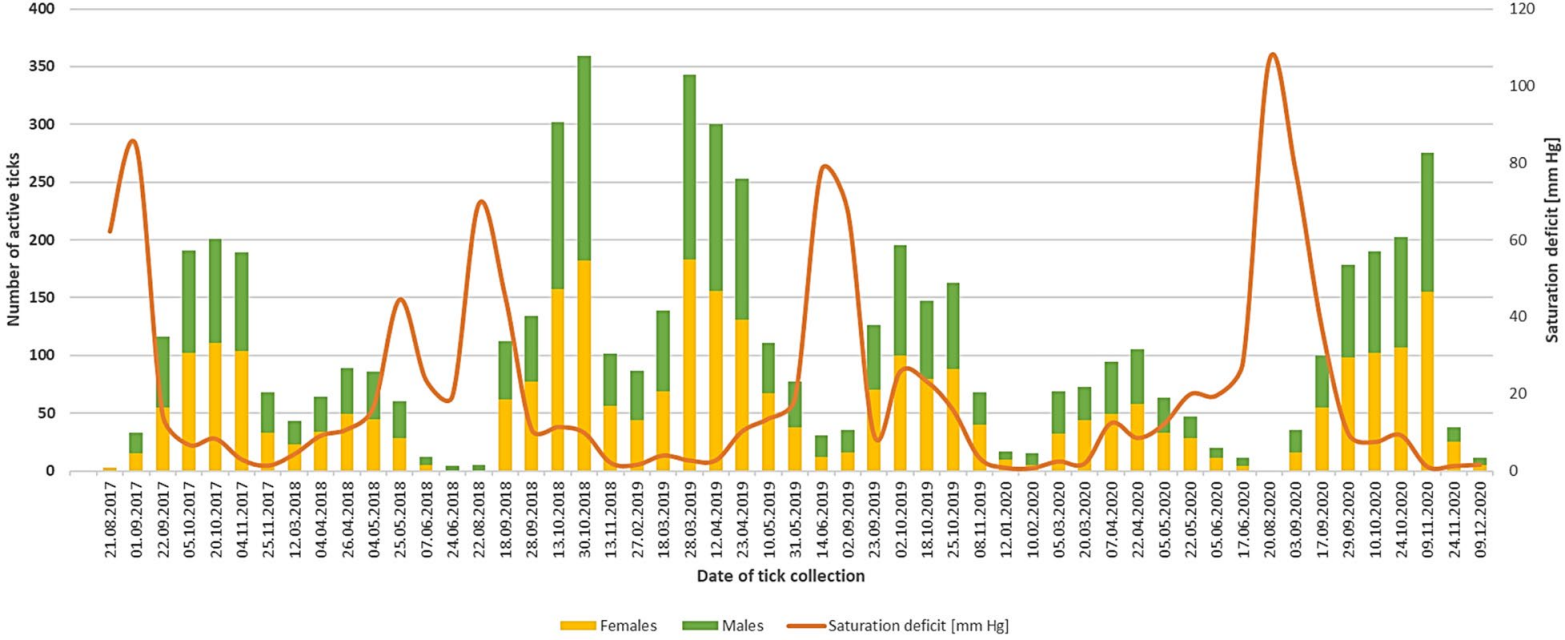
Seasonal activity and abundance of *D. reticulatus* in eastern Poland.

In 2020, due to the mild and mostly frost-free and snowless winter, active t**i**cks were collected throughout the year, excluding the summer months (diapause period) (Fig. 2). The activity of *D. reticulatus* in 2020 differed statistically significantly compared to 2019 (Z = − 2.2219, p = 0.0264). In 2019 (with an anomaly of + 12 days with snow cover in January and anomaly of the mean air temperature of + 3.32 °C in February and + 2.75 °C in March compared to the long-term average), there was a clear statistically significant difference in the number of active ticks in spring over the number of active ticks in autumn (Z = 2.1721 p = 0.030) (Figs. 1, 2). In the other years of the study, a predominance of the autumn peak was observed. The rhythms of the seasonal activity of *D. reticulatus* did not differ significantly throughout the study period (H = 5.1958, p = 0.0744).

*D. reticulatus* ticks were collected in the temperature range of 4.0–24.0 °C and relative air humidity of 32.4–90.6% (Supplementary Table S1). The greatest numbers of adult *D. reticulatus* ticks were active in the air temperature range of 10.0–18.0 °C. An increase in the temperature above 20.0 °C was accompanied by a decrease in the number of active ticks. The greatest numbers of *D. reticulatus* specimens were collected in the relative air humidity range of 60.0–70.0%, whereas a decrease in the number of active ticks was observed at a humidity value below 50% (Supplementary Table S1).

**Figure 3:**
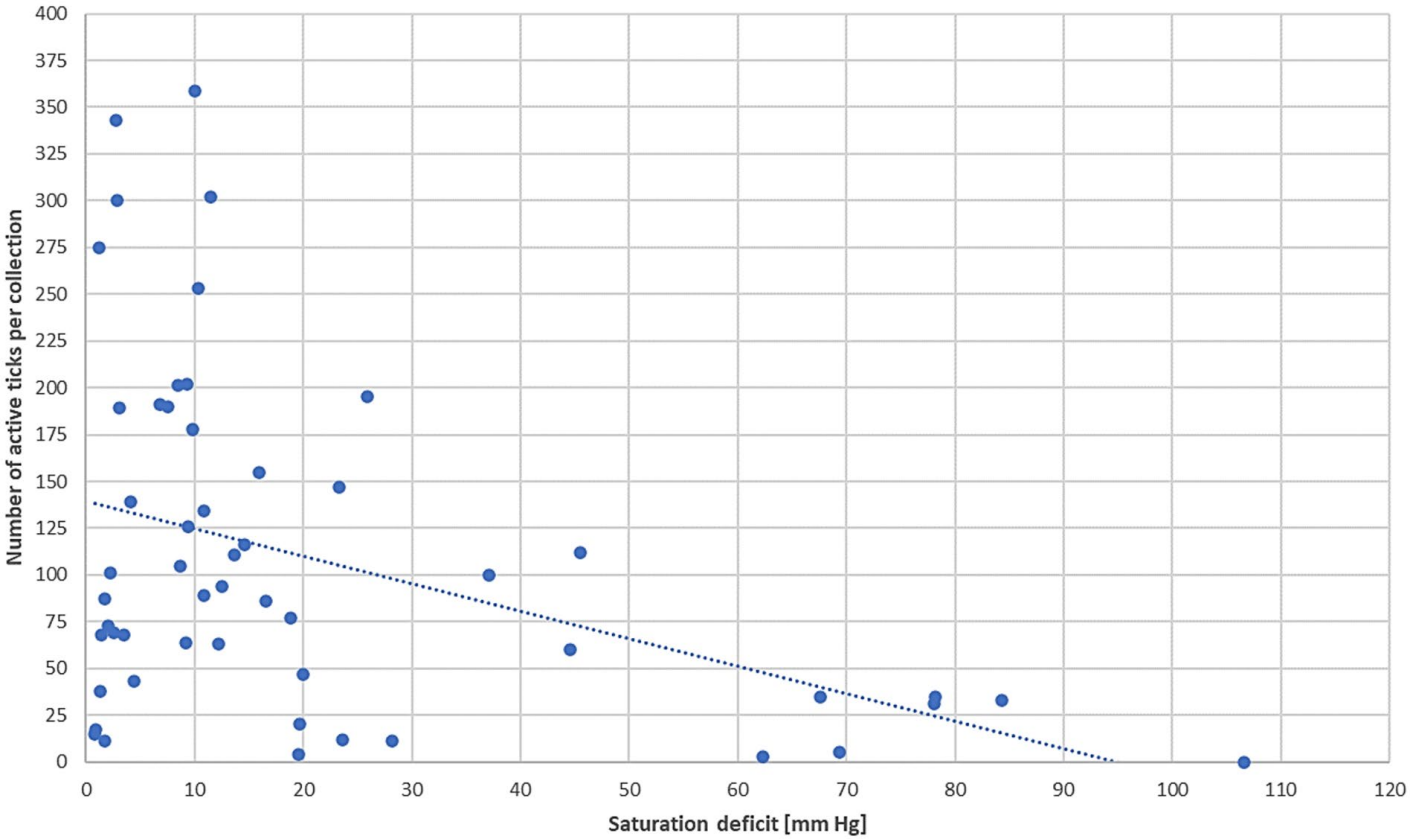
Correlation between the value of the saturation deficit and the number of active *D. reticulatus* ticks.

*D. reticulatus* ticks were collected in a saturation deficit range of 0.90–106.62 mmHg (Fig. 2, Supplementary Table S1). As the saturation deficit increased, the number of active *D. reticulatus* ticks clearly declined. This regularity was observed in each year of the study (Fig. 2). The highest number of ticks showed activity with saturation deficiency up to 30 mmHg (Fig. 3), which significantly influences the activity of *D. reticulatus* (r_s_ = − 0.3135, p = 0.0222).

## Discussion

As highlighted by many authors, progressing climate changes are the cause of changes in the number and range of many animal species, including ticks^22,30,31^. The increase in the number of local populations of these arthropods observed in recent years in many European countries, e.g. in the Baltic states^32^, Poland^19–21,33^, and Germany^34,35^, or the emergence of new species of ticks in areas previously considered tick-free^36,37^ contributes to an increase in the epidemic threat and the risk of transmission of new or sporadically existing tick-borne pathogens^38,39^.

Our results show that, in eastern Poland in 2017–2020, there was a statistically significant increase in the mean, mean maximum, and mean minimum air temperature compared to the long-term period, especially in the summer and autumn months (Table 1, Fig. 1). Climate warming also resulted in high negative anomalies in the number of days with snow cover (− 70 in 2020) (Fig. 1). Such conditions should be considered favorable for the development of *D. reticulatus*. Extension of the growing season results in a longer period of the activity of potential tick hosts, allowing ticks to complete their development cycle within one year^38,40^.

Due to the prolonged period of positive mean air temperatures during the late autumn months with no deviation of the mean long-term total precipitation from the norm (Table 1, Fig. 1), the juvenile forms of *D. reticulatus* that are particularly vulnerable to adverse weather changes find optimal conditions for development, increasing their chance to develop into adulthood before winter. Our previous research shows that, at this stage, 67.9% females and 60.0% males survive in adverse winter conditions^41^. Nevertheless, the activity of *D. reticulatus* adults during the winter months was also reported in other studies, also those conducted during the snow cover period^42,43^.

During our research, we observed the activity of *D. reticulatus* in the temperature range of 4.0–24.0 °C, with most specimens showing activity at the temperature of 10.0–18.0 °C (Fig. 2, Supplementary Table S1). Temperature is one of the most important factors influencing the host-seeking activity of *D. reticulatus*^24^. Active adult *D. reticulatus* specimens were found in the field at a temperature close to the ground of − 0.1 °C^44^. In laboratory conditions, these ticks are able to survive thermal shock even at − 20 °C^10^. The range of temperatures at which we collected *D. reticulatus* is similar to the temperatures at which this species of ticks was collected in the region and other parts of Poland^45–47^. However, it differs from the thermal preferences of *D. reticulatus* in the British Isles, where the activity of this species ceases when the air temperature exceeds 15 °C over consecutive 5 days^10^. In contrast, in eastern Poland, ticks of this species were collected at the temperature of 31.0 °C^46^.

The results of our research also indicate the saturation deficit as a factor that significantly influences the activity of *D. reticulatus*. Most ticks of this species show activity when the value of this coefficient is up to 30 mmHg (Fig. 3). *D. reticulatus* ticks have a narrower tolerance range in terms of saturation deficiency than *I. ricinus*, which in eastern Poland show the greatest activity with saturation deficiency up to 60 mmHg, while active specimens of this species were also collected at a saturation deficiency of 120 mmHg^48^. This dependence may be related to the fact that *D. reticulatus* ticks have a greater proportion of fat bodies in relation to body weight than *I. ricinus*; therefore, specimens at the oldest physiological age do not need to be active in unfavorable conditions to collect host's blood^49,50^.

The population of *D. reticulatus* studied by us showed a variable distribution of peaks of seasonal activity, depending on the year. More *D. reticulatus* ticks were active in spring in 2019 and in autumn in the other years of observation (Fig. 2, Supplementary Table S1). This relationship is not fully explained. Previous long-term studies on the activity of this species in eastern Poland indicated a clear advantage of the autumn peak^45,46,51^. However, a similar rhythm of seasonal activity of *D. reticulatus* was also observed in other parts of the region in 2019^21^. Predominance of spring activity peak over autumn was reported in the population of *D. reticulatus* from north-eastern Poland^52^ and the European part of Russia^53^. Similar rhythms of seasonal activity of *D. reticulatus* were reported by Szymański^54^. The research conducted by the author over 30 years ago in northern Poland indicated a predominance of spring and autumn peaks. In this part of the country, the spring activity of *D. reticulatus* started in mid-March and lasted until mid-June, with the peak activity noted in April. The autumn season of *D. reticulatus* activity lasted from mid-August to the end of November, with a peak in mid-October.

In the temperate climate zone, the temperature in the winter months exceeding the long-term mean may be a determinant of changes in tick activity peaks^55^. In our opinion, this phenomenon in the studied area does not result directly from the local weather conditions, but is determined by a number of other not yet studied factors, including the availability of hosts and the impact of climatic conditions on their abundance.

In conclusion, the deficit saturation exerts an impact on the activity of adult *D. reticulatus* ticks. Ticks of this species may also be active during winter months at positive values of air temperature and in the absence of snow cover. The studied *D. reticulatus* population is characterized by high plasticity in terms of weather conditions. It should be expected that the progressive climate change will not have a negative impact on the rhythms of the seasonal activity of *D. reticulatus* but may contribute to an increase in the population size of this species.

## Methods

### Weather conditions

Data from the Meteomodel website compiled on the basis of data published by the Institute of Meteorology and Water Management in Warsaw were used to analyze the weather conditions^26^. The data were provided by a meteorological station located nearest to the site of the field research plot (Lublin Radawiec, 51.2174 °N, 22.3927 °E), 30 km in a straight line.

The analysis of the weather conditions included the following parameters (for each month): mean air temperature, mean maximum and mean minimum air temperature, number of days with maximum temperature below 0 °C, total precipitation, mean relative air humidity, and number of days with snow cover. The data covered the years in which the field research was conducted, i.e. 2017, 2018, 2019, and 2020. Additionally, the average values of these parameters for the reference period covering the 30 years preceding the start of the research, i.e. 1987–2016, were calculated. On this basis, the values of anomalies were calculated for each year in relation to the reference period.

### Study area

**Figure 4:**
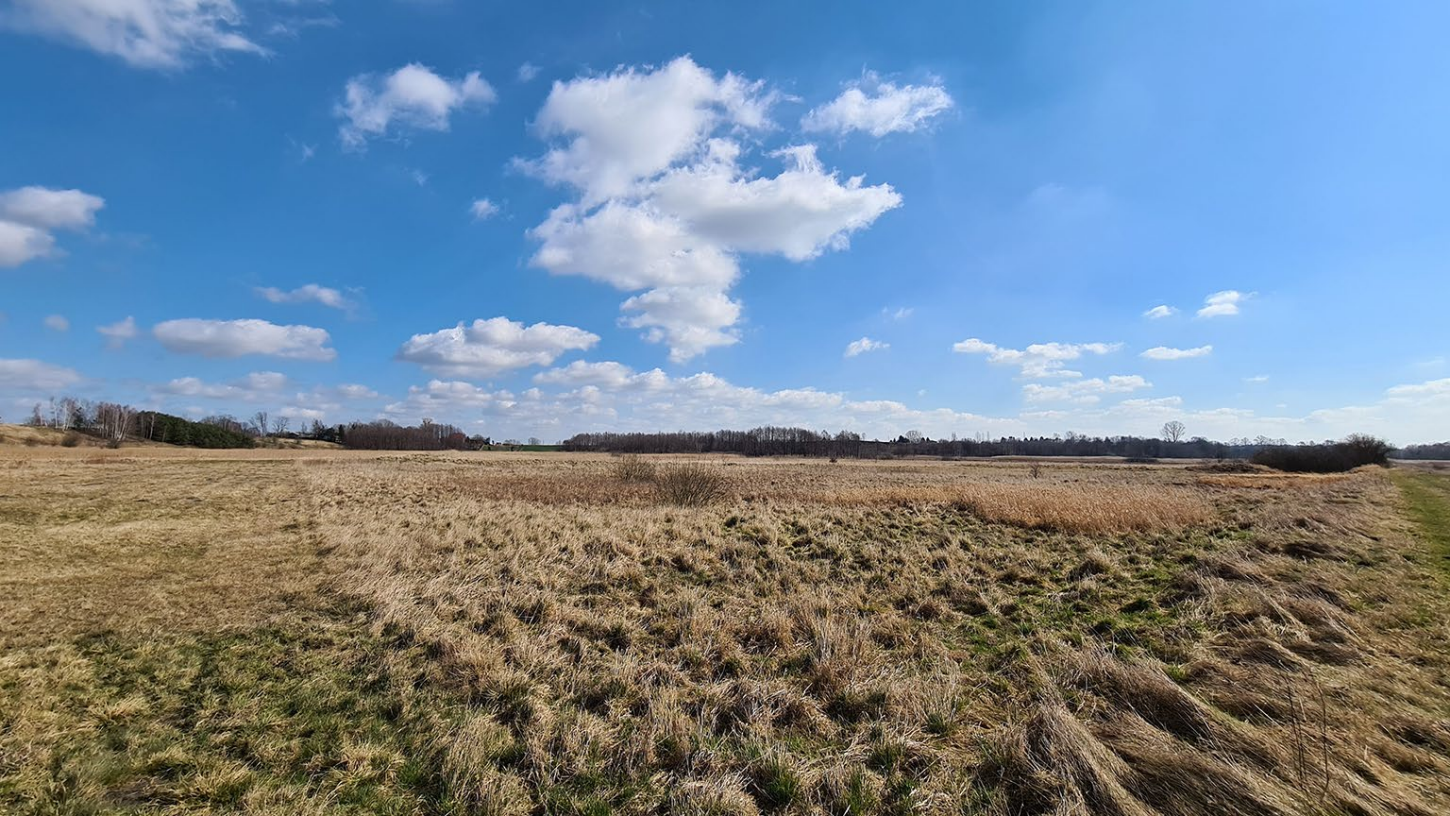
Meadow with the established research plot. Photo by Z. Zając.

The field studies on the occurrence and activity of *D. reticulatus* ticks were carried out in eastern Poland (51.3555 °E, 22.7595 °N) in 2017–2020 in a habitat regarded as preferred by this species^4^. The research plot was established within an unused agricultural meadow with the dominance of Molinio-Arrhenatheretea vegetation at the initial stage of ecological succession, surrounded by meadows, fallow land, and a watercourse (Fig. 4).

### Tick surveillance

Ticks were collected using the flag method consisting in sweeping a white flannel cloth over vegetation. After about 5 m, the sheet was turned over and attached ticks were transferred with the use of metal tweezers into a 50 cm^3^ container, in which blades of grass were placed to ensure the optimum level of humidity. Ticks were collected within 30 min. The collection was made between 10:00 and 11:00.

As a rule, the field research was carried out at two-week intervals (due to unfavorable weather conditions, e.g. rainfall or strong wind, this date had to be postponed). The collection of ticks was not carried out in the summer months (July-mid-August) when adults of this species enter behavioral diapause and adult specimens remain inactive in eastern Poland^45,46^ and in the period when there was snow cover and/or negative air temperature.

Each time during the field collection, the current weather conditions, i.e. temperature and relative air humidity, were measured using the Data Logger R6030 device (Reed Instruments, Wilmington, NC, USA). The measurements were used to calculate the saturation deficit according to the formula^56^.

$$StDf=(1-\frac{RH}{100})\times {4.9463\times e}^{0.0621\times T}$$

where: StDf—saturation deficit; RH—relative humidity; e—actual vapor pressure; T—temperature.

In the laboratory, the species, sex, and developmental stage of the collected specimens were identified^57^. Next, the individuals were frozen at − 80 °C (ULTF miller, Arctico, Esbjerg, Denmark) until further analysis.

### Statistical analysis

The type of distribution of the obtained data was checked using the Shapiro Wilk test. The ANOVA test was used to check the significance of differences in weather conditions between the years of the study and the reference period.

The statistical significance of differences in the number of active ticks between all study years was verified with the Kruskal–Wallis test. The Mann–Whitney U test was used to compare the significance of differences in the number of active ticks between two years/seasons. The influence of saturation deficiency on the activity of *D. reticulatus* was tested using the rho-Spearman correlation.

The value of *p* < 0.05 was considered statistically significant. Statistical calculations were performed using the STATISTICA 11 PL statistical package (StatSoft, TIBCO Software Inc, Palo Alto, CA, USA).

## Supplementary Information

Supplementary Table 1.
