# Supplementary material for: Seasonal activity of Dermacentor reticulatus ticks in the era of progressive climate change in eastern Poland
Source: Sci Rep. 2021 Oct 14;11:20382. doi: 10.1038/s41598-021-99929-y (PMC8516878; doi:10.1038/s41598-021-99929-y)
Supplement: Supplementary file 1 — Supplementary Table 1. [file 41598_2021_99929_MOESM1_ESM.pdf]

Date of collection	T [ºC]	RH [%]	StDf [mm Hg]	Number of collected ticks		
				Females	Males	Total
21.08.2017	22.3	55	62.31	3	0	3
01.09.2017	23.7	50.6	84.32	15	18	33
22.09.2017	15.5	59	14.61	55	61	116
05.10.2017	11.8	66.5	6.84	102	89	191
20.10.2017	13.3	69	8.44	111	90	201
04.11.2017	7	70	3.05	104	85	189
25.11.2017	4	80.3	1.45	33	35	68
12.03.2018	10.5	75.2	4.43	23	20	43
04.04.2018	13.9	70.2	9.23	34	30	64
26.04.2018	14.4	66.3	10.89	49	40	89
04.05.2018	16	52.9	16.59	45	41	86
25.05.2018	20.9	50	44.57	28	32	60
07.06.2018	18	44.2	23.6	5	7	12
24.06.2018	16.9	47	19.54	0	4	4
22.08.2018	23.5	40	69.4	0	5	5
18.09.2018	21	50.1	45.48	62	50	112
28.09.2018	14	61	10.88	77	57	134
13.10.2018	14.2	59	11.51	157	145	302
30.10.2018	13.5	60.8	9.98	182	177	359
13.11.2018	6.5	77.8	2.21	56	45	101
27.02.2019	5.5	80.9	1.69	44	43	87
18.03.2019	10.5	77.7	4.07	69	70	139
28.03.2019	8.9	80.5	2.8	183	160	343
12.04.2019	8	76.6	2.85	156	144	300
23.04.2019	14.5	70	10.38	131	122	253
10.05.2019	15	55.5	13.68	67	44	111
31.05.2019	16.9	40.7	18.86	38	39	77
14.06.2019	24	40	78.07	12	19	31
02.09.2019	22.9	46.6	67.64	16	19	35
23.09.2019	13	58.2	9.42	70	56	126
02.10.2019	18.3	55	25.91	100	95	195
18.10.2019	17.8	50	23.34	80	67	147
25.10.2019	16	60.3	15.91	88	75	155
08.11.2019	8	70.5	3.46	40	28	68
12.01.2020	4	88	0.9	10	7	17
10.02.2020	5	90.6	0.81	5	10	15
05.03.2020	8.1	80	2.54	32	37	69
20.03.2020	7.7	83	2.06	44	29	73
07.04.2020	16	75	12.49	49	45	94
22.04.2020	15	80	8.65	58	47	105
05.05.2020	15	66.2	12.19	33	30	63
22.05.2020	17	55	19.97	28	19	47
05.06.2020	16.9	50	19.66	11	9	20
17.06.2020	19	42.3	28.21	4	7	11
20.08.2020	26.3	32.4	106.62	0	0	0
03.09.2020	24	40.5	78.19	16	19	35
17.09.2020	20	54.7	37.14	55	45	100
29.09.2020	13.8	66	9.81	98	80	178
10.10.2020	12	62	7.58	102	88	190
24.10.2020	13	59	9.35	107	95	202
09.11.2020	8.8	77.9	1.19	155	120	275
24.11.2020	6	86	1.35	25	13	38
09.12.2020	7.2	85	1.71	5	6	11
Total				3072	2718	5782

Supplementary Table 1. Total number of collected *D. reticulatus* ticks and weather parameters during field research, T – temperature, RH – relative humidity, StDf – saturation deficit .
